# Value of Tips in Shoeing1Read before the Pennsylvania State Veterinary Medical Association, Cresson, September 3, 1895.

**Published:** 1895-11

**Authors:** Chas. Bland

**Affiliations:** Roxborough, Philadelphia, Pa.


### VALUE OF TIPS IN SHOEING.[1]

BY CHAS. BLAND, V.S.,
ROXBOROUGH, PHILADELPHIA, PA.

THE subject matter of this report is one that comes under "shoeing," and in this case refers to the value of "tips." A horse I have been driving for nearly three years, about twenty-five years of age, stumbled very much when I bought him, and through that fault had met with an accident and was knee-sprung. During the previous summer he had trotted in 2.40. I did not change the style of shoeing that year. The following summer I thought I would try tips. I did, and found great improvement both in his gait and also his legs. In the winter, owing to the steep hills and the slippery state of the roads, I am obliged to have full sharpened shoes on him, then by the spring he stumbles and gets shaky again.

He will carry the fore tips eight weeks and the hind ones six weeks. I never have them taken off so long as they are fast enough for safe driving. The weight of the shoe is thirteen

[1] Read before the Pennsylvania State Veterinary Medical Association, Cresson, September 3, 1895.

ounces. I travel on all kinds of roads — Belgian blocks, asphalt, cobble-stones, and rough and smooth country roads. The heel of his foot expands about half an inch during the summer and contracts during the winter. I use the tip the same thickness all around and have it let in the wall, so it leaves the quarters and frog flush with the tips.

I have had several horses suffering from navicular disease shod this way, and always found more or less improvement according to how far the disease had advanced. I can recall one case where, after the change in shoeing, the horse travelled fairly well. It is well to remember that it will not suit all; also, that marked results must not be looked for the next day. On the contrary, they will sometimes be worse for a few days after the change.

